# Enniatin and Beauvericin Biosynthesis in *Fusarium* Species: Production Profiles and Structural Determinant Prediction

**DOI:** 10.3390/toxins9020045

**Published:** 2017-01-25

**Authors:** Vania C. Liuzzi, Valentina Mirabelli, Maria Teresa Cimmarusti, Miriam Haidukowski, John F. Leslie, Antonio F. Logrieco, Rocco Caliandro, Francesca Fanelli, Giuseppina Mulè

**Affiliations:** 1 Institute of Sciences of Food Production, CNR, 70126 Bari, Italy; vania.liuzzi@ispa.cnr.it (V.C.L.); teresa.cimmarusti@ispa.cnr.it (M.T.C.); miriam.haidukowski@ispa.cnr.it (M.H.); antonio.logrieco@ispa.cnr.it (A.F.L.); giuseppina.mule@ispa.cnr.it (G.M.); 2 Institute of Crystallography, CNR, 70126 Bari, Italy; valentina.mirabelli@ic.cnr.it; 3 Department of Economics, University of Foggia, 71121 Foggia, Italy; 4 Department of Plant Pathology, Kansas State University, Manhattan, 66506 KS, USA; jfl@ksu.edu

**Keywords:** enniatin, beauvericin, ESYN1, mycotoxins, *Fusarium*, homology modelling, multivariate analysis, backbone angles

## Abstract

Members of the fungal genus *Fusarium* can produce numerous secondary metabolites, including the nonribosomal mycotoxins beauvericin (BEA) and enniatins (ENNs). Both mycotoxins are synthesized by the multifunctional enzyme enniatin synthetase (ESYN1) that contains both peptide synthetase and S-adenosyl-l-methionine-dependent *N*-methyltransferase activities. Several *Fusarium* species can produce ENNs, BEA or both, but the mechanism(s) enabling these differential metabolic profiles is unknown. In this study, we analyzed the primary structure of ESYN1 by sequencing *esyn1* transcripts from different *Fusarium* species. We measured ENNs and BEA production by ultra-performance liquid chromatography coupled with photodiode array and Acquity QDa mass detector (UPLC-PDA-QDa) analyses. We predicted protein structures, compared the predictions by multivariate analysis methods and found a striking correlation between BEA/ENN-producing profiles and ESYN1 three-dimensional structures. Structural differences in the β strand’s Asn789-Ala793 and His797-Asp802 portions of the amino acid adenylation domain can be used to distinguish BEA/ENN-producing *Fusarium* isolates from those that produce only ENN.

## 1. Introduction

**Figure 1:**
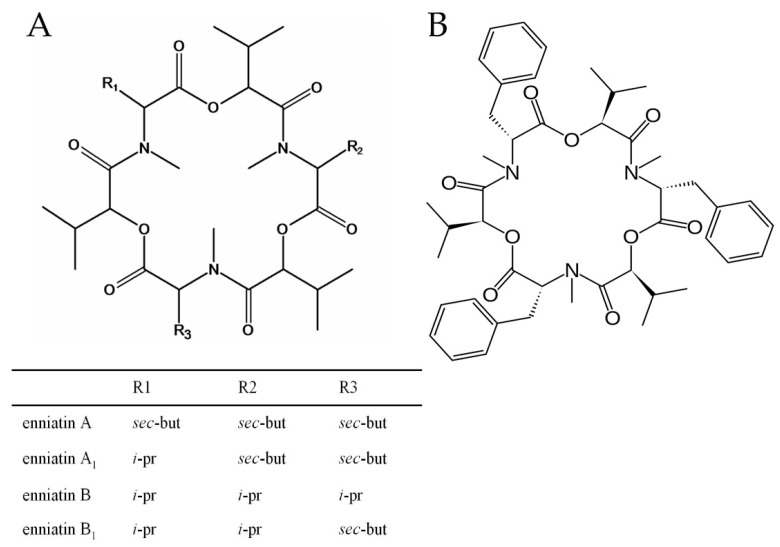
Enniatin (**A**) and beauvericin (**B**) chemical structures.

Enniatins (ENNs) and beauvericin (BEA) are structurally-related mycotoxins (Figure 1) produced by several fungal species. These compounds have antibiotic and ionophoric properties and different bioactivities [1].

Structurally, BEA and ENNs are non-ribosomal cyclic hexadepsipeptides that consist of alternating d-2-hydroxyisovaleric acid and *N*-methyl l-amino acids. In beauvericin, the three amino acid residues are aromatic *N*-methyl-phenylalanines, whereas in type A and B enniatins, the amino acid residues are aliphatic *N*-methyl-valine, or *N*-methyl-isoleucine, or mixtures of these amino acids [2]. The subunits are linked by peptide bonds and intramolecular ester (lactone) bonds, forming a cyclic depsipeptide.

Beauvericin was first isolated from the entomopathogenic fungus *Beauveria bassiana* [3]. It has antibiotic, antifungal, insecticidal and cancer cell antiproliferative and anti-haptotactic (cell motility inhibitory) activities in vitro [4].

The first enniatin to be described was enniatin A (ENN A) in 1947 by Gäumann et al. [5], as a metabolite produced by the fungus *Fusarium orthoceras* var. *enniatinum* with toxicity toward bacteria, fungi and plant shoots [6]. ENNs are mainly produced by filamentous fungi in the genus *Fusarium*, although they have been isolated from a few other fungal genera, such as *Verticillium hemipterigenum* [7] and *Halosarpheia* spp. [8].

To date, 29 naturally-occurring enniatin analogues have been identified. The variants most frequently detected in foods and feeds, especially in cereals, are enniatin A, A_1_ (ENN A_1_), B (ENN B), B_1_ (ENN B_1_) and B_4_ (ENN B_4_), together with smaller amounts of enniatins C, D, E and F. Structural differences related to the *N*-methyl-amino acid are responsible for the different bioactivities of these mycotoxins [9].

Nonribosomal peptide synthesis occurs through a mechanism of multistep condensation catalyzed by non-ribosomal peptide synthetases (NRPSs) [10]. These multi-enzyme complexes are modularly organized in domains, with highly conserved core motifs and specific catalytic activities [11]. Nonribosomal synthesis of peptides requires at least three domains: (i) an adenylation (A)-domain for amino acid recognition and activation; (ii) a peptidyl carrier protein (PCP)-domain that transports units of the activated intermediate [12]; and (iii) a condensation domain that catalyzes peptide bond formation [13].

In *Fusarium*, ENNs production is catalyzed by the non-ribosomal multifunctional enzyme, enniatin synthetase (ESYN1) [14,15,16,17], through the condensation of three dipeptidol units followed by cyclization. This enzyme, encoded by the *esyn1* gene, consists of one 347-kilodalton polypeptide chain. It was purified and characterized by Zocher and coworkers [14,18,19] from *Fusarium equiseti* (ex. *scirpi*). It synthesizes ENNs from their primary precursors, i.e., valine, leucine or isoleucine, d-2-hydroxyisovaleric acid and *S*-adenosylmethionine. Its multifunctional structure is composed of different domains: two adenylation domains, some peptidyl carrier protein domains, a methyltransferase domain and three condensation domains. The two adenylation domains are responsible for the activation of the substrates d-2-hydroxyisovaleric acid and l-amino acid as acyl adenylate intermediates.

Data on the occurrence of BEA and ENNs in food and feed have recently been reported by the European Food Safety Authority (EFSA) [20] in the first risk assessment related to these two mycotoxins. In cereals, beauvericin and enniatins often co-occur, as do enniatins A, A_1_, B and B_1_, the four ENNs most frequently detected in food and feed. High co-occurrence levels are expected as the two mycotoxin groups are structurally related, may be produced by the same *Fusarium* species and are products of the same metabolic pathway [21,22]. Indeed, some *Fusarium* spp. produce BEA, ENNs or both, with the mechanisms responsible for the end product variation and their regulation remaining unknown.

The biosynthesis of secondary metabolites is regulated by genetic, epigenetic and environmental factors. The interaction of these factors determines the type and amount of mycotoxin(s) produced and varies by strain [23,24]. The emerging importance of BEA and ENNs in cereal contamination and the recent study of their toxic effect(s) on human and animal health, together with the possibility that these compounds may be used as pharmaceutical products [1,25], require more detailed knowledge of the molecular mechanism(s) underlying their biosynthesis in toxigenic fungal species.

In this study, we evaluated the production of BEA and ENNs by strains in several *Fusarium* species grown under the same experimental conditions. Our working hypothesis was that differences in the production profiles depended on the ESYN1 protein sequences and three-dimensional structure. We used a bioinformatics approach, based on molecular modelling, comparative analysis of protein sequences and multivariate analysis of structural models, to identify the structural determinants responsible for the differential biosynthesis of BEA and ENNs biosynthesis. This study provides critical information on the regulation of the production of these emerging mycotoxins.

## 2. Results

### 2.1. Analytical Method

**Figure 2:**
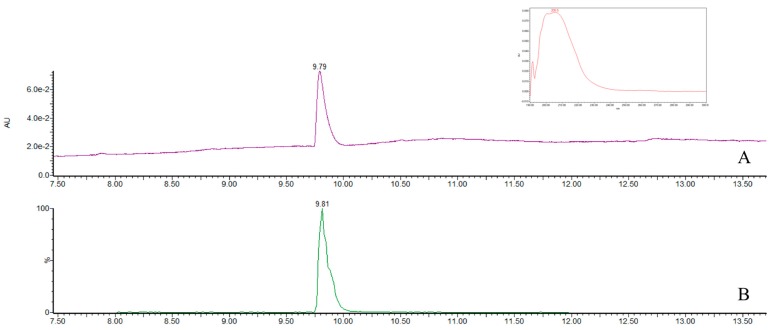
Chromatograms of beauvericin standard (0.4 µg/mL) in UPLC/PDA (**A**) and in SIR (**B**). In the box on the upper right, the typical ultraviolet (UV) spectrum of the standard solution is shown.

**Figure 3:**
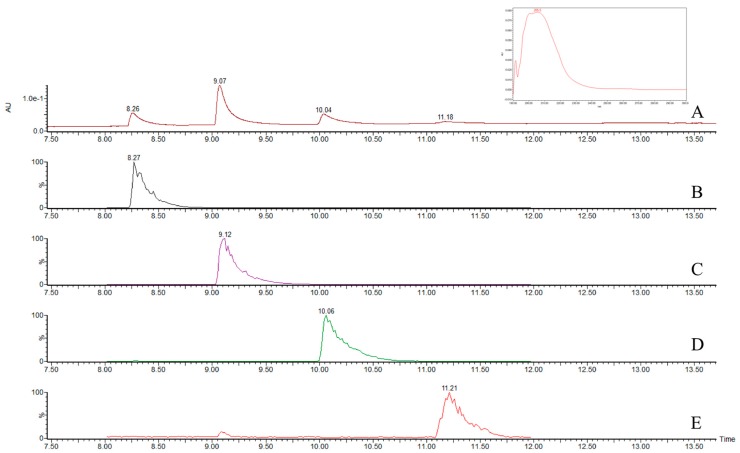
UPLC/PDA chromatogram of enniatin standards in UPLC/PDA (**A**); SIR of ENN B (7 µg/mL) (**B**), ENN B_1_ (20 µg/mL) (**C**), ENN A_1_ (8 µg/mL) (**D**) and ENN A (1.2 µg/mL) (**E**). In the box on the upper right, the typical UV spectrum of enniatins at 205 nm is shown.

Mean recoveries of fortified agar plugs (*n* = 3) at levels of ENN A (0.3–0.6 µg/g), ENN A_1_ (2.0–4.0 µg/g), ENN B (1.9–3.8 µg/g), ENN B_1_ (5.4–10.8 µg/g) and BEA (2.0–4.0 µg/g) were respectively 80.6%, 81.5%, 78.3%, 84.0% and 86.7% with relative standard deviations of 3.3%, 2.6%, 5.2%, 7.6% and 6.8%, respectively. Figure 2 shows the chromatograms of beauvericin standard in UPLC/PDA and in single ion recording (SIR). Figure 3 shows the chromatogram of combined standard solutions of the four ENNs evaluated in UPLC/PDA and in SIR.

The limit of quantification (LOQ) values were calculated according to *s*/*n* = 10 (Table 1). The linearity of the standard curves at three determinations of six concentration levels was between 0.9988 and 0.9997. LOD was calculated as LOQ/3.

### 2.2. Enniatins and Beauvericin Production by Fusarium Isolates

The chemotype of *Fusarium* isolates known from previous publications or as determined by chemical analysis is reported in Table 2. The amount of ENNs and BEA produced in chemically-defined production medium (FDM) and potato dextrose agar (PDA) are indicated in Tables S1 and S2. All of the strains used produced quantifiable amounts of ENNs, with higher amounts produced on PDA than on FDM. The chemotype profile of each strain was the same in both media. All *F. fujikuroi* strains and *F. proliferatum* strain KSU 4854 produced both ENNs and BEA (Figure S1). The other *F. proliferatum*, strain KSU 830, produced only ENN A, ENN B and ENN B_1_. *Fusarium avenaceum* strains produced only ENN A and ENN B [26]. Strains ITEM 3403 and 3404 were used as positive controls for the chemical analyses and could produce all of the ENNs and BEA (Tables S1 and S2). They were selected from the Agri-Food Toxigenic Fungi Culture Collection of the Institute of Sciences of Food Production, Italian National Council of Research (CNR), Bari (www.ispa.cnr.it/Collection). They were identified according to the criteria and synoptic keys of Nelson et al. [27] and by molecular analysis. The ability of *Fusarium avenaceum* to produce both BEA and ENNs has been previously reported [28,29].

ENN A, ENN B and ENN B_1_ were all produced by *F. andiyazi* KSU 4647, *F. thapsinum* KSU 4093 (Tables S1 and S2) and by all of the *F. verticillioides* strains. As an example, Figure S1 shows well-resolved chromatograms of ENNs and BEA from the *F. proliferatum* KSU 4854 FDM culture.

### 2.3. Sequences Analysis

The *esyn1* sequences of the 13 *Fusarium* isolates were extracted from the published and unpublished genomes by BLASTN search by querying with the available sequences of *F. equiseti* (ex *scirpi*) (NCBI Accession Number Z18755.3) and *F. proliferatum* (NCBI Accession Number JF826561.1). The protein sequences were predicted using the Sequence Translation tools of the EMBOSS Programs (EMBL-EBI), manually curated with exon/intron boundary predictions from SpliceView (http://bioinfo4.itb.cnr.it/) and confirmed by RT-PCR of different partial sequences (Multifasta S1 and S2).

The *esyn1* mRNA from the *F. verticillioides* strain FGSC 7600 did not contain the 568-bp intron predicted by Ma et al. [32] (Ensembl Fungi Accession Number FVEG_09993). This intron was predicted incorrectly because the sequence was partially mapped on two different contigs with a region missing. This intron also was missing in all of the other sequences included in this study. The *F. proliferatum* transcripts lacked the 134-bp intron reported by Zhang et al. [33]. The *esyn1* mRNA from KSU 3089G had a 13-bp duplicated insertion that results in a premature stop and the synthesis of a truncated protein (Multifasta S2).

**Figure 4:**
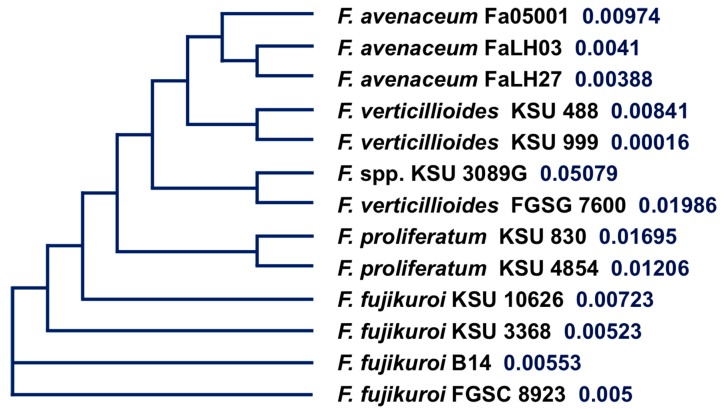
Cladogram of the 13 *Fusarium* ESYN1 sequences produced by the algorithm Phylogenetic Tree and based on the multialignment constructed using the Clustal Omega 1.2.1 Multiple Sequence Alignment Tool (http://www.ebi.ac.uk/Tools/msa/clustalo/). Values after each strain indicate ML Bayesian posterior bootstrap probability.

The protein tree inferred from the Clustal Omega multi-alignment of the 13 amino acid sequences (Figure 4) separates the *F. fujikuroi* and *F. proliferatum* groups and the other species.

### 2.4. Structural Analysis

#### 2.4.1. Structural Models’ Comparison

**Figure 5:**
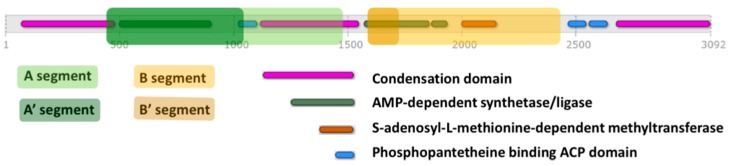
Schematic representation of functional domains of ESYN1 from *Fusarium proliferatum* (UniProt Entry G3GBU7). The A, A′, B and B′ segments considered in this study for the comparative analysis are highlighted (see the text for details).

A preliminary Principal Component Analysis (PCA) was performed on data matrices formed by the segment A and segment B regions of the 13 ESYN1 sequences. Analysis of the loadings (Figures S2 and S3) identified regions with minimal deviation, including the first 600 residues of segment A and the first 115 residues of segment B. Thus, we restricted our analysis to these regions, hereafter referred to as segments A′ and B′ (Figure 5).

**Figure 6:**
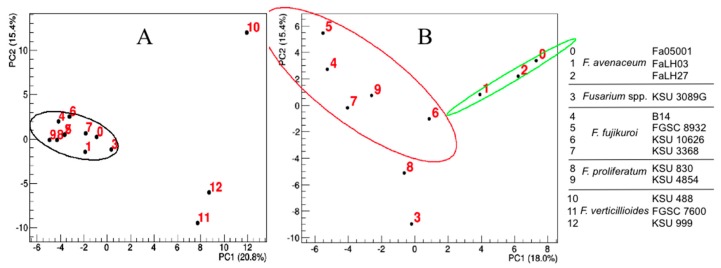
Score plots of the first two principal components obtained after the application of PCA to the A′ segment of (**A**) all 13 ESYN1 sequences of *Fusarium* strains and of (**B**) the subset constituted by removing the *Fusarium verticillioides* strains (Samples 10, 11, 12). The percentage of data variance explained by each principal component is reported on the corresponding axis labels. The 95% confidence level ellipses indicate the results of hierarchical clustering.

Protein Angular Value (PAV) profiles calculated for the 13 A′ segments (Figure S4A) were processed by PCA (Figure 6). Sequences (10, 11 and 12) from the *F. verticillioides* strains have unique structural motifs that distinguish them from the other ten ESYN1 sequences (Figure 6A). To improve the resolution and to enable a more informative exploration of the remaining gene sequences, these three sequences were removed from the dataset, and the PCA was run again (Figure 6B). In this second analysis, the first principal component explains 18% of the variance and generates two clusters and two distinct values (three and eight). One cluster, sequences 0, 1 and 2, contains sequences from the strains of *F. avenaceum*. The sequences in the other cluster (4, 5, 6, 7 and 9) are from strains that produce both enniatins and beauvericin (Table S2). Thus, structural determinants in the A′ segment suffice to explain the specific features of the *F. verticillioides* and *F. avenaceum* enzymes and, more importantly for demonstrating our working hypothesis, to identify ESYN1 sequences that enable the production of both ENNs and BEA. Similar results were obtained when the B′ fragment was analyzed (Figure S5). With the B′ segment, however, the differentiation of the enzyme classes is not as clear, with sequence 10 not being separate from the main cluster and the sequences from enzymes producing both BEA and ENNs not as well separated from the others.

**Figure 7:**
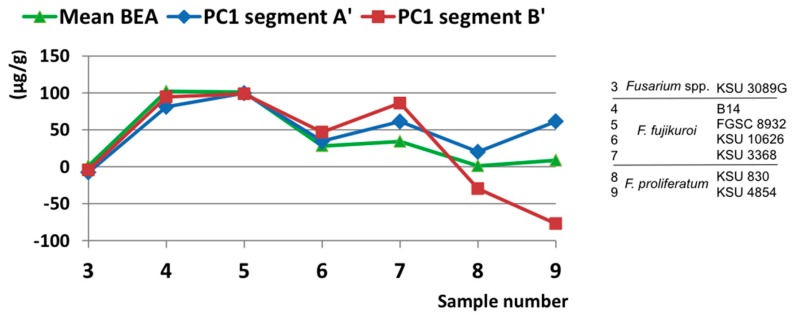
Beauvericin (BEA) production (green) as a function of PC1 calculated for fragments A′ (blue) and B′ (red). PC1 scores were rescaled to overlap with measured quantities: for fragment A′, values were multiplied by −7 and added to 50, and for fragment B′, values were multiplied by 240 and added to 30.

Comparing in vitro BEA production with structural differences through multivariate analysis results in a more quantitative assessment of the distinctions identified in the PCA analysis (Figure 7). BEA production profiles and PC1 scores are highly correlated, with a Pearson’s correlation coefficient of 0.83 for the A′ segment values and 0.80 for the B′ segment. The original PC1 scores are in arbitrary units and were rescaled to overlap with the production values.

#### 2.4.2. Structural Interpretation of Multivariate Analysis Results

In addition to enabling qualitative comparison of structural models, PCA can also provide information about the residues responsible for sample separation, through the analysis of the loadings. When the loadings, appropriately rescaled, are plotted on the top of the score plots of Figure 6 (Figure S6), the largest loadings, positive or negative, are those responsible for separating the data points representing the ESYN1 structural models. Since in Figure 6 the data points are mainly separated along the first principal component (*X*-axis of the plots), residues with the largest PC1 loadings are those relevant for our analysis. Table 3 reports a list of hotspot residues more clearly singled out by such a criterion. The residues responsible for grouping the data points are located in well-defined regions of the structural models, and involve contiguous β strands or secondary structural elements coupled to a nearby loop.

**Figure 8:**
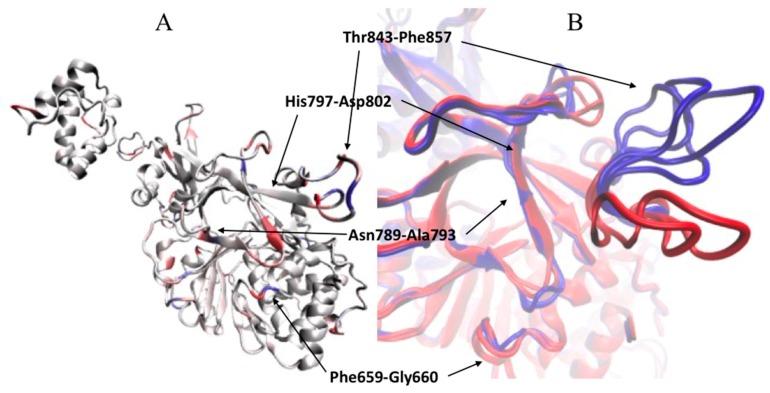
(**A**) Structural model of the A′ fragment of *Fusarium avenaceum*, strain Fa05001, in cartoon representation. Residues are colored based on loading values (red: high positive value; blue: high negative value; white: low value) of the first principal component. (**B**) Overlap of the structural models of the A′ fragment of *Fusarium fujikuroi* strains B14 and FGSC 8932 (red) and *Fusarium* spp., strain KSU 3089G, *Fusarium fujikuroi*, strain KSU 10626, *Fusarium proliferatum*, strain KSU 830 (blue). Red models, corresponding to Samples 4 and 5, are associated with higher production of BEA, and blue models, corresponding to Samples 3, 6 and 8, are associated with no BEA production.

The significance of residues in BEA production by *F. fujikuroi* and *F. proliferatum* can be seen in Figure 8A, where residues are colored based on their loading value. These residues all occur in two contiguous β sheets of the A′ fragment, His797-Asp802 and Ala793-Asn789, and in the nearby loop containing residues Phe659 and Gly660. The corresponding conformational changes (Figure 8B) of the enzymes from the BEA-producing strains differentiate them from the non-producing strains. The β strand’s Asn789-Ala793 and His797-Asp802, and the nearby loop, Thr843-Phe857, also are involved in separating the *F. avenaceum* sequences from the other sequences, indicating the key role played by this structural region in the chemotype expressed by different *Fusarium* strains.

## 3. Discussion

We developed a useful method that uses a photodiode array and MS-QDa detectors in a unique chromatographic run to confirm and quantify ENN and BEA production. The UPLC system is faster than HPLC, and the MS-QDa detector confirms the analyte. We used an extraction procedure without a sample clean-up step and obtained chromatograms that could be used to quantify the toxins. This process avoids the risk of unexpected co-elutions and provides analytical confidence for the mass detection.

BEA and ENNs have a wide range of biological activities, including insecticidal, antifungal, antibiotic and cytotoxic properties. They also have been evaluated as enzyme inhibitors, particularly towards acyl-CoA, with BEA the most potent cholesterol acyltransferase inhibitor of microbial origin [34]. The biggest interest in BEA and ENNs is related to their cytotoxicity and anticancer properties, as a mixture of ENNs induces apoptosis in several human cancer cell lines at low concentrations [35], and ENN A_1_ and B_1_ induce apoptotic cell death and disrupted the extracellular regulated protein kinase (ERK) activity associated with cell proliferation [36]. The potential pharmaceutical and biotechnological applications of these molecules require deeper and more detailed knowledge of their biosynthesis. In particular, a better understanding is needed of both ENNs and BEA synthesis, both individually and as mixtures of BEA and various ENN analogues. This information also is required to assess the risks posed by the presence of the producing *Fusarium* species as natural contaminants of cereal grains and their by-products and in the development of risk assessment maps.

The chemical structure of BEA and ENNs confers specific biological activities to them [37,38]. There are five synthetic steps leading to the formation of these cyclic hexadepsipeptides, all catalyzed by the multi-modular enzyme ESYN1: (1) activation as acyl adenylate intermediates of branched-chain amino acids and d-2-hydroxyisovaleric acid by two adenylation domains; (2) transfer of amino- and hydroxyacyl residues to specific thiol groups; (3) methylation of thioesterified amino acids; (4) condensation reactions between the thioesterified *N*-methyl amino acids and d-2-hydroxyisovaleric acid; and (5) elongation and cyclization of depsipeptides.

Zocher and coworkers postulated that different cyclic hexadepsipeptides are formed due to the low substrate specificity of the enzyme toward branched-chain amino acids and to the relative abundance of the enzyme’s substrates in the cellular pool [14]. Studies of substrate specificity found that phenylalanine could be replaced by numerous aromatic or aliphatic amino acids, including β-phenylserine, ortho-, meta-, para-fluoro-phenylalanine, isoleucine, norleucine and leucine [39].

Different *Fusarium* strains, however, preferentially incorporate some amino acids regardless of the external substrate pool, and some ENNs can be isolated reliably from particular species or strains. For example, *Fusarium sambucinum* preferentially produces ENN A [5], and *Fusarium scirpi* preferably synthesizes ENN B [40]. Previously reported data on the production of BEA/ENN by *F. verticillioides* are ambiguous. Confusion results from intra-specific variation within *F. verticillioides* and from the subdivision of the former *F. moniliforme* into multiple species, one of which is *F. verticillioides* [41]. Many of the older studies use only the *F. moniliforme* name, and it often is not possible to determine whether the strains evaluated were *F. verticillioides* or another species that also was included in *F. moniliforme* at the time the work was published. In most of these cases, clarifying the true species identify often is difficult, if not impossible, as the strains used in the studies were not from standard public culture collections, such as the strains in this study are. All of the strains used in this study were selected because the strains and published/verified genome sequences from them were available for identification and the production of both BEA and/or ENNs could be tested experimentally.

None of the strains we examined produced only BEA, so our *de facto* goal was to pinpoint the factors discriminating the production of BEA/ENN from the production of ENN only. Thus, all of the strains were cultured on common culture medium under the same environmental conditions and phenotypes assigned to genetic determinants, while interference associated with substrate availability was minimized.

Although epigenetic and environmental factors are important in the regulation of secondary metabolite production, genetic determinants play critical roles, as well. The most obvious genetic determinants are those that encode the enzyme(s) that synthesize the metabolite in question. Genes that encode other proteins, e.g., proteins that transport metabolites into the cell from the external environment or that synthesize precursor metabolites, also may be important in determining which metabolites are available for the biosynthetic process. For ESYN1, the substrate binding pocket of the amino acid-adenylation domain may be responsible for enzyme substrate specificity. A 10-amino acid signature sequence in the acid-adenylation domain of different cyclic hexadepsipeptide synthetases alleles from 11 different fungal species [42] may be responsible for the proposed amino acid specificity.

The 13 allelic *Fusarium esyn1* sequences used in this work were taken from published and unpublished genomes and confirmed by RT-PCR. These sequences were not always the same as the predicted sequences available in the public databases. For example, we did not confirm the presence of a 568-bp intron in *esyn1* from the *F. verticillioides* strain FGSC 7600 predicted by the Ensembl fungi database (FVEG_09993). Since the sequence in the database mapped partially on two non-adjacent contigs, the intron was probably predicted incorrectly by Ma et al. [32]. This hypothesis was further confirmed by the absence of this intron in all of the other strains evaluated in this study. Furthermore, the presence of a 134-bp intron unique to the published *F. proliferatum esyn1* sequence from Zhang et al. [33] was not seen in the transcripts from any of the *F. proliferatum* strains included in this study, revealing the absence of introns. The ESYN1 protein from strain KSU 3089G lacks the last 628 amino acids of the protein, due to the insertion of a 13-bp duplicated segment that generates a premature stop codon. As this strain produces ENN A, B and B_1_, the N-terminal portion of the protein sequence is not essential for the synthesis of any of these metabolites.

The clustering inferred from the comparison of the 13 amino acid sequences (Figure 4) distinguishes the *F. fujikuroi* and *F. proliferatum* group from the other strains of the species we evaluated. This separation was expected given the different chemotypes of these two groups. The separation of ENN producers from BEA/ENN producers thus was consistent with the differences observed in the three-dimensional structural analysis. To compare structural models, the three-dimensional protein models were reduced to one-dimensional profiles and these values then compared with multivariate analysis methods. The sequence clustering based on structural characteristics separated the *F. fujikuroi* strains and *F. proliferatum* KSU 4854 from the other strains, which mirrors the metabolite production chemotypes (Figure 6). The first group of strains produced both BEA and ENNs, while the second group produced only ENNs. Residues responsible for ESYN1 structural differences amongst *Fusarium* strains were identified based on PCA scores and loadings and those potentially involved in BEA production identified. The most striking results come from residues on the same β sheet and in nearby loops. Thus, a limited and well-defined structural region along with the residues potentially required for BEA biosynthesis have been identified. Allelic variation in this region suffices to explain the ability to synthesize BEA and is associated with different metabolic profiles of the strains that carry the different alleles. The region of interest is part of the amino acid-adenylation domain of the protein, which determines which amino acids are activated and bound to the nascent peptide.

Important residues in the region that activates the hydroxy acid (d-hydroxy isovalerate) also were identified. Variation at these residues should not have a great impact on the metabolic profile, as this precursor is a building block for BEA and all ENNs.

## 4. Conclusions

In summary, we confirmed our working hypothesis that the difference in ENN and BEA chemotypes of the strains under study depends on the ESYN1 protein sequence and its three-dimensional structure. Allelic variation in the amino acid-adenylation domain is associated with the different chemotypes observed. The novel analytical method in which UPLC was coupled with an MS-QDa detector resulted in a unique chromatographic run that could measure BEA and ENN levels in agar samples. Such a methodology is essential for any effort to select strains for higher levels of metabolite production or for testing fungal isolates recovered from contaminated substrates for their production capabilities. We evaluated only 13 strains and cannot generalize our conclusions to the entire *Fusarium* genus, but have validated this approach for future studies on the relationship between enzyme structure and function. Furthermore, the new knowledge about enzyme regulation and metabolite detection is an important advance in our ability to understand the promises and hazards posed by beauvericin and enniatins.

## 5. Materials and Methods

### 5.1. Fungal Strains

The strains used in this study are listed in Table 2. They were selected based on the availability of genomic information and the strain availability for chemical and molecular analysis. KSU 488, KSU 999, KSU 830 and KSU 4854 strains were identified based on genomic sequencing (unpublished data), confirming previous identifications by other authors [43,44,45]. *Fusarium* spp. KSU 3089G may be an inter-specific hybrid between *F. fujikuroi* and *F. proliferatum* based on cross fertility and phylogenetic analyses of amplified fragment length polymorphisms (AFLPs) and DNA sequencing (J.F. Leslie, personal communication).

Strains were grown for 5 days at 25 °C on PDA (Oxoid, Rodano (MI), Italy). Conidia were harvested, and a conidial suspension was prepared in sterilized distilled water. Conidia were counted in a Thoma chamber, and the suspension was diluted to a final concentration of 10^6^ conidia/mL. For each experiment, 50 μL of the concentration-adjusted conidial suspension were inoculated at single point agar plates.

### 5.2. Media and Growth Conditions

The media used in this study were PDA and an enniatin-inducing medium, FDM agar medium [46,47,48] containing the following per liter of distilled water: 12.5 g of glucose, 4.25 g of NaNO_3_, 5 g of NaCl, 2.5 g of MgSO_4_·7H_2_O, 1.36 g of KH_2_PO_4_, 10 mg of FeSO_4_·7H_2_O and 2.9 mg of ZnSO_4_·7H_2_O. Experiments were performed in triplicate. A conidial suspension of each strain was inoculated on 9-cm Petri dishes containing 30 mL of either PDA or FDM agar medium. The inoculated plates were incubated at 25 °C for 14 days in the dark.

For molecular analysis, the agar medium was overlaid with a sterile cellophane sheet prior to inoculation to facilitate the removal of fungal biomass for RNA extraction.

### 5.3. Chemicals and Preparation of Standards

All solvents (HPLC grade) were purchased from VWR International Srl (Milan, Italy). Ultrapure water was produced by a Millipore Milli-Q system (Millipore, Bedford, MA, USA). ENN and BEA standards (purity >99%) were supplied by Sigma-Aldrich (Milan, Italy). Standard stock solutions of a mixture of ENNs (A 3% = 0.03 mg/mL, A_1_ 20% = 0.2 mg/mL, B 19% = 0.19 mg/mL and B_1_ 54% = 0.54 mg/mL) and BEA (1 mg/mL) were prepared by dissolving the solid commercial toxin standards in methanol. Adequate amounts of the stock solution were dried under a nitrogen stream at 50 °C and reconstituted with methanol-water (70:30, *v*/*v*). Standard solutions for UPLC calibration were prepared by using different concentrations within an appropriate calibration range (Table 1). Standard solutions were stored at −20 °C and warmed to room temperature prior to use.

### 5.4. Determination and Confirmation of Enniatins (A, A_1_, B and B_1_) and Beauvericin from Agar Medium

The potential of a subset of 13 *Fusarium* spp. isolates to produce ENNs and BEA in vitro was evaluated by ultra-performance liquid chromatography coupled with a photodiode array and Acquity QDa mass detector (UPLC-PDA-QDa). Five grams of agar culture material for each culture were extracted with 10 mL of methanol on an orbital shaker for 60 min. Six milliliters of the extract were evaporated to dryness under a stream of nitrogen at 50 °C. The residue was dissolved in 500 µL of methanol/water (70:30, *v*/*v*) and filtered through a 0.20-µm regenerated cellulose filter (Grace Davison Discovery Science). Ten microliters of the extract were injected into the full loop injection system of a UPLC^®^ system Waters Acquity (Milford, MA, USA), equipped with an ESI interface, and with a binary solvent manager, a sample manager, a column heater, a photodiode array and QDa detectors.

The analytical column was an Acquity UPLC BEH C18 (2.1 × 100 mm, 1.7 μm) preceded by an Acquity UPLC^®^ in-line filter (0.2 µm). The temperature of the column was set at 50 °C. The flow rate of the mobile phase was set at 0.35 mL/min. The toxins were determined in both detectors, i.e., the PDA set at 205 nm, and, after the effluent, into the ESI interface, without splitting. The mobile phase consisted of a binary gradient applied as follows: the initial composition (50% (A) water-0.1% formic acid/50% (B) acetonitrile-0.1% formic acid) was kept constant for 2 min, then Solvent B was increased linearly up to 75% in 8 min, then linearly increased up to 80% in 2 more min and, finally, kept constant for 4 min. For column re-equilibration, Eluent B was decreased to 50% in 1 min and kept constant for 4 min.

For LC/MS analyses, the ESI interface was used in positive ion mode, with the following settings: desolvation temperature 600 °C; capillary voltage 0.8 kV, sampling rate 5 Hz. The mass spectrometer was operated in full scan (600–800 *m*/*z*) and in single ion recording (SIR) mode, by monitoring the individual masses of each compound (enniatins: A 682 *m*/*z*, A_1_ 668 *m*/*z*, B 640 *m*/*z*, B_1_ 654 *m*/*z*; and beauvericin: 784 *m*/*z*). MassLynx^®^ 4.1 mass spectrometry software was used for data acquisition and processing. Retention time for enniatins B, B_1_, A_1_, A were about 8.5, 9.2, 10.2 and 11.4 min, respectively, and beauvericin about 9.8 min. Toxins were quantified by measuring peak areas and comparing these values with a calibration curve obtained from standard solutions.

### 5.5. Sequence Data Analysis

Enniatin synthetase gene sequences were extracted from published or unpublished genomes (Table 2) by BLASTN search (http://blast.ncbi.nlm.nih.gov/Blast.cgi) with the available sequences ESYN1 from *F. equiseti* (ex. *scirpi*) (CAA79245.2) and FpBEAS from *F. proliferatum* (AEN14638.1) used as the search terms. Protein sequences were predicted by the Sequence Translation Tools of the EMBOSS programs (EMBL-EBI) and manually curated. Exon/intron boundary prediction was made by SpliceView (http://bioinfo4.itb.cnr.it). Predicted protein sequences were confirmed by RT-PCR.

### 5.6. RNA Isolation and Reverse Transcription

Fungal biomass was removed from cellophane-sheet overlays on FDM agar medium for RNA isolation. Total RNA was isolated by using the RNeasy Plant Mini Kit (Qiagen, Hilden, Germany), according to the manufacturer’s instructions, and stored at −80 °C. RNase-free DNAse I (Qiagen) was used to remove contaminating genomic DNA. First strand cDNA was synthesized with an RT Omniscript Reverse Transcription kit (Qiagen). Reactions contained 2 μg of total RNA, 2 μL of oligo d(T)_16_ (10 μM), 2 μL of 10× RT-PCR buffer, 2 μL dNTPs (10 mM), 1 μL (4 U) of RNAse inhibitor, 1 μL (4 U) of Omniscript Reverse Transcriptase and RNase-free water to a final volume of 20 μL. cDNA synthesis reactions were performed at 37 °C for 1 h.

### 5.7. RT-PCR and Sequencing

Primers were designed with the Primer3 web interface Version 0.4.0 [49] on the basis of multialignment of the sequences obtained from the BLASTN search in order to amplify different regions of the transcripts (Table 3). PCR reactions were performed as follows: 50 ng of cDNA, dNTPs 0.2 mM, 5 U of 5PRIME HotMaster Taq DNA Polymerase, 2 μL of 5PRIME 10× HotMaster Taq Buffer with magnesium, forward and primer reverse 300 nM each (Table 4) and H_2_O to the final volume of 20 μL. The amplification products were purified with the enzymatic mixture EXO/SAP (exonuclease I, *Escherichia coli*/shrimp alkaline phosphatase (Thermo Fisher Scientific Inc., Waltham, MA, USA). DNA of both strands was purified by gel filtration through Sephadex G-50 (Amersham Pharmacia Biotech, Cologno Monzese (MI), Italy), sequenced with a Big Dye Terminator Cycle Sequencing Ready Reaction Kit (Applied Biosystems, Foster, CA, USA), and analyzed on an “ABI PRISM 3730 Genetic Analyzer” (Applied Biosystems, Foster, CA, USA).

### 5.8. Multi-Sequence Alignment

The Clustal Omega (ClustalO) 1.2.1 Multiple Sequence Alignment Tool (EMBL-EBI) was used to align the 13 ESYN1sequences from the different *Fusarium* species. A cladogram of the 13 sequences was obtained by using the Phylogenetic Tree algorithm, implemented in the ClustalO package.

Based on the multiple sequence alignment, the whole sequence of each protein was split into two segments, termed “A” and “B”, which removes long gaps at the beginning, at the end and in the middle of the alignment. The placement of the two segments was based on the *F. proliferatum* cyclic peptide synthetase (UniProt Entry G3GBU7, Figure 5). For the structural analysis, asterisks in position 378 of Fv_0488 (VLA*YPS) and Fv_0999 (VLA*YPS), indicating stop codons probably generated by sequencing errors (Multifasta S2), were substituted with the conserved tryptophan (W).

### 5.9. Structural Model Generation

For each protein, a structural model for the entire sequence was generated by the RaptorX Protein Structure Prediction web server (http://raptorx.uchicago.edu/StructurePrediction/). Structural models with exactly the same number of residues, particularly suited for comparative analysis, were then obtained by considering structural predictions within segments A and B, and by deleting unaligned residues. All editing operations on structural models were performed by using Visual Molecular Dynamics (VMD) scripts [50].

### 5.10. Structural Models Comparisons

Largely distant structural models cannot be compared by using standard procedures based on Cartesian coordinates. For example, the calculation of the root mean squared distance (RMSD) requires that the best superposition of two models be found, before the average distance between corresponding C_α_ atoms is calculated. Superimposing one structure on another is difficult when the relative orientation of consecutive domains is different. To overcome this problem, we compared the structural models by using internal coordinates, namely the backbone dihedral angles of single residues. This process sidesteps the overlap step, and the models can then be compared by considering differences in their dihedral angles. A residue-by-residue profile of internal coordinates is obtained by calculating the PAV, defined as:

(1) $$PAV_{i}=\frac{180}{\pi }\cos{}^{-1}\left(\cos(\psi_{i}+\varphi_{i})\right)$$

where ψ*_i_* and ϕ*_i_* are the backbone dihedral angles of the *i*-th residue. PAV values range between 0° and 180° and ψ*_i_* + ϕ*_i_* values expressed in degrees, while avoiding the problem of range definition connected with the circular nature of the angular variables ψ*_i_* and ϕ*_i_*. Equation (1) was derived based on arguments contained in Caliandro et al. [51]. PAV values were calculated by using an adapted version of the T-PAD tool. A data matrix was formed for the PAV profiles calculated within the A and B fragments of each of the ESYN1 sequences. This matrix is based on aligned residues and does not contain gaps since the columns corresponding to the gaps were deleted in the structural model generation step.

Structural comparison was performed by PCA, an unsupervised multivariate analysis method that requires minimal experimental information, does not suffer from model bias and provides objective classification criteria. PCA is a projection method for visualizing complex data by reducing the dimensionality of the dataset [52]. The data matrix is decomposed into a number of principal components (PCs) that maximize the explained variance in the data with each successive component. PCs are calculated as eigenvectors of the covariance matrix of the data. PAV profiles can be projected into a new coordinate system, where the PCs form the axes. These score plots can be used to discern patterns in the data and to cluster ESYN1 sequences. Coordinates of the PC’s eigenvalues on the original axes are called loadings and carry information about the specific residues responsible for the clustering. PCA is followed by a hierarchical clustering step, performed on representative points in the space of the selected principal components (score plots). The 95% confidence level ellipses are calculated for clusters containing more than three representative points. Structural comparison calculations were made with the RootProf program [53].

## Figures and Tables

**Table 1:** Enniatin and beauvericin experimental parameters, Limit of Quantification (LOQ) of the analytical method and single ion recording (SIR).

Mycotoxin	Elemental Formula [M + H]^+^	Retention Time (min)	SIR (*m/z*)	Calibration Range (µg/mL)	LOQ UPLC/QDa (µg/g)
Enniatin A	C_36_H_63_N_3_O_9_	11.4	682	0.09−1.20	0.015
Enniatin A_1_	C_35_H_61_N_3_O_9_	10.2	668	0.30−8.00	0.05
Enniatin B	C_33_H_57_N_3_O_9_	8.5	640	0.04−7.60	0.07
Enniatin B_1_	C_34_H_59_N_3_O_9_	9.2	654	0.04−21.60	0.07
Beauvericin	C_45_H_57_N_3_O_9_	9.8	784	0.02−40.00	0.04

**Table 2:** Enniatin (ENN) and beauvericin (BEA) production profile of *Fusarium* strains.

Species	Strain	Metabolic Profile	Genome Accession/Reference	ESYN1 CDS Accession
*Fusarium fujikuroi*	B14	ENN A, ENN B, ENN B_1_, BEA	ANFV01 [30]	-
*Fusarium fujikuroi*	FGSC 8932	ENN A, ENN B, ENN B_1_, BEA	FRVF01 [31]	KY026610
*Fusarium fujikuroi*	KSU 10626	ENN A, ENN B, ENN B_1_, BEA	FRVG01 [31]	KY026609
*Fusarium fujikuroi*	KSU 3368	ENN A, ENN B, ENN B_1_, BEA	FRVH01 [31]	KY026611
*Fusarium verticillioides*	FGSC 7600	ENN A, ENN B, ENN B_1_	AAIM02 [32]	KY026615
*Fusarium verticillioides*	KSU 488	ENN A, ENN B, ENN B_1_	this study	-
*Fusarium verticillioides*	KSU 999	ENN A, ENN B, ENN B_1_	this study	KY026616, KY026617
*Fusarium* spp.	KSU 3089G	ENN A, ENN B, ENN B_1_	this study	KY026614
*Fusarium proliferatum*	KSU 830	ENN A, ENN B, ENN B_1_	this study	KY026613
*Fusarium proliferatum*	KSU 4854	ENN B, ENN B_1_, BEA	this study	KY026612
*Fusarium avenaceum*	Fa05001	ENN A *, ENN B *	JPYM01 [26]	-
*Fusarium avenaceum*	FaLH03	ENN A *, ENN B *	JQGD01 [26]	-
*Fusarium avenaceum*	FaLH27	ENN A *, ENN B *	JQGE01 [26]	-

* [26].

**Table 3:** Residues with the largest loadings, identified as responsible for clustering the ESYN1 sequences. Residue names and numbers are based on the ESYN1 sequence of *F. avenaceum*, strain Fa05001.

Hotspot Residues	Secondary Structure	Description
*Ala502-Val503*	β strand	Discrimination of *F. verticillioides* strains in A′ segment
*Ala505-Trp510*	β strand	
*Gln725-Phe730*	α helix	Discrimination of *F. avenaceum* strains in A′ segment
*Ser749-Asn753*	α helix	
*Asn789-Ala793*	β strand	
*His797-Asp802*	β strand	
*Thr843-Phe857*	Loop	
*Asn1744*	β strand	Discrimination of *F. avenaceum* strains in B′ segment
*Phe1746*	Loop	
*Phe659-Gly660*	Loop	Explanation of beauvericin metabolic profiles of *F. fujikuroi* and *F. proliferatum* strains in A′ segment
*Asn789-Ala793*	β strand	
*His797-Asp802*	β strand	
*Thr843-Phe857*	Loop	
*Met1760-Gly1764*	α helix	Explanation of beauvericin metabolic profiles of *F. fujikuroi* and *F. proliferatum* strains in B′ segment
*Leu1817*	β strand	
*G1u822*	Loop	

**Table 4:** Primers and annealing temperatures used in the RT-PCR reactions.

Species	Primer forward	Primer reverse	T annealing
*F. fujikuroi*	ACTGTTGCGTTGACTTCCAA	ACAAGTTCACCAATTGCCCC	54 °C
*F. proliferatum*	TTTCTCATGGCTGCTGGAGA	GGTTATCATTCGCGTCACCC	55 °C
*Fusarium *spp. KSU 3089G	GAGCCGTGCATCTCTTTCTG	CTTTCACAGTGACGCGAACA	54 °C
*F. verticillioides*	GGTCGTCGCTTCAATGCTAG	GAACTCTCGCTCTGACCGTA	54 °C
*F. verticillioides*	CGCAATCGGTGAACTTGTGA	GGCCAACAATTCGCTACCAA	54 °C

## Supplementary Materials

The following are available online at www.mdpi.com/2072-6651/9/2/45/s1. Figure S1: UPLC/PDA chromatogram of the agar extract from the sample *Fusarium proliferatum* KSU 4854 grown on chemically-defined production medium (FDM). (**A**) SIR of ENN B (0.058 µg/g), (**B**) ENN B_1_ (0.3 µg/g) and (**C**) BEA (4.8 µg/g). Figure S2: (**A**) Loading plot obtained by processing the protein angular value (PAV) profiles of the segment A of the ESYN1 sequences of the 13 *Fusarium* isolates, with the selected region of minimal variations highlighted. (**B**) Overlap of the corresponding structural models of segment A, performed according to the positions of the Cα atoms lying in the selected region (highlighted). Figure S3: (**A**) Loading plot obtained by processing the PAV profiles of segment B of the ESYN1 sequences of the 13 *Fusarium* isolates, with the selected region of minimal variations highlighted. (**B**) Overlap of the corresponding structural models of segment B, performed according to the positions of the Cα atoms lying in the selected region (highlighted). Figure S4: PAV profiles calculated for the ESYN1 sequences of the 13 *Fusarium* isolates, shown separately for segment A’ (left) and B’ (right). The samples’ numbering follows that indicated in Table 2. (**B**) Overlap of the corresponding structural models of segment B, performed according to the positions of the Cα atoms lying in the selected region (highlighted). Figure S5: Score plots of the first two principal components (PC1 and PC2) obtained by applicating principal component analysis (PCA) to the segment B’ of (**A**) all 13 ESYN1 sequences of *Fusarium* isolates and of (**B**) the subset constituted by eliminating the *Fusarium verticillioides* strains (Samples 10, 11, 12). The percentage of data variance explained by each principal component is reported on the corresponding axis labels. The 95% confidence level ellipses indicate the results of hierarchical clustering. Figure S6: Score (red dots) and loading (blue dots) plots of the first two principal components obtained after application of PCA to the segment A’ of (**A**) all 13 ESYN1 sequences of *Fusarium* isolates and of (**B**) the subset constituted by eliminating the *Fusarium verticillioides* strains (Samples 10, 11, 12). The percentage of data variance explained by each principal component is reported on the corresponding axis labels. The residue number of the loadings more distant from the (0, 0) point is reported. Table S1: Enniatin and beauvericin production of *Fusarium* strains on chemically-defined production medium (FDM). Table S2: Enniatin and beauvericin production of *Fusarium* strains on potato dextrose agar (PDA) medium.
