# Supplementary material for: Enniatin and Beauvericin Biosynthesis in Fusarium Species: Production Profiles and Structural Determinant Prediction
Source: Toxins (Basel). 2017 Jan 25;9(2):45. doi: 10.3390/toxins9020045 (PMC5331425; doi:10.3390/toxins9020045)
Supplement: Supplementary file 1 [file toxins-09-00045-s001.pdf]

Supplementary Materials: Enniatin and Beauvericin Biosynthesis in *Fusarium* Species: Production Profiles and Structural Determinant Prediction

Vania C. Liuzzi, Valentina Mirabelli, Maria Teresa Cimmarusti, Miriam Haidukowski, John F. Leslie, Antonio F. Logrieco, Rocco Caliandro, Francesca Fanelli and Giuseppina Mulè

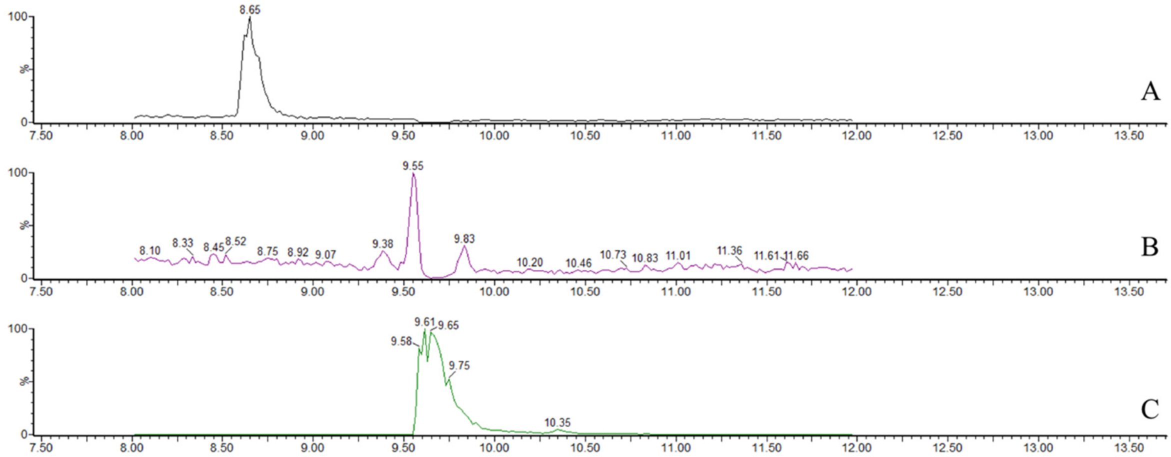

Figure S1. UPLC/PDA chromatogram of the agar extract from the sample *Fusarium proliferatum* KSU 4854 grown on chemically-defined production medium (FDM). (**A**) SIR of ENN B (0.058 µg/g), (**B**) ENN B_1 (0.3 µg/g) and (**C**) BEA (4.8 µg/g).

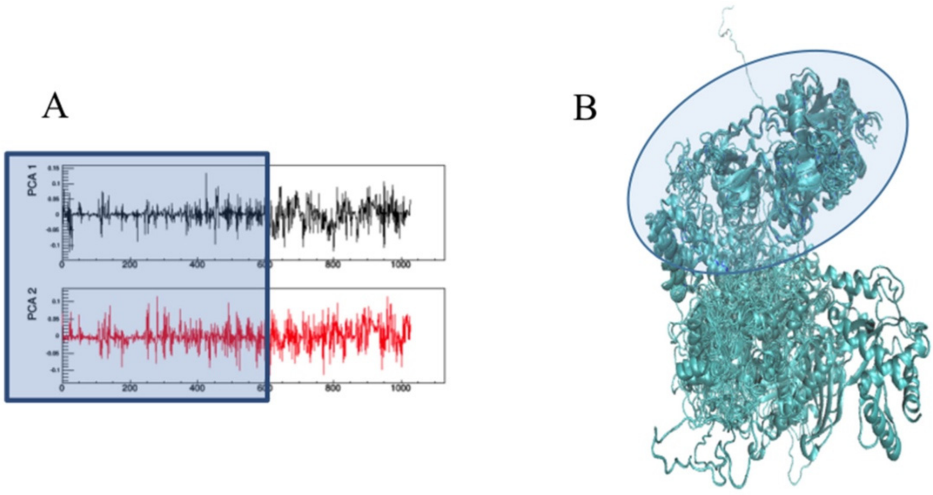

Figure S2. (**A**) Loading plot obtained by processing the protein angular value (PAV) profiles of the segment A of the ESYN1 sequences of the 13 *Fusarium* isolates, with the selected region of minimal variations highlighted. (**B**) Overlap of the corresponding structural models of segment A, performed according to the positions of the Cα atoms lying in the selected region (highlighted).

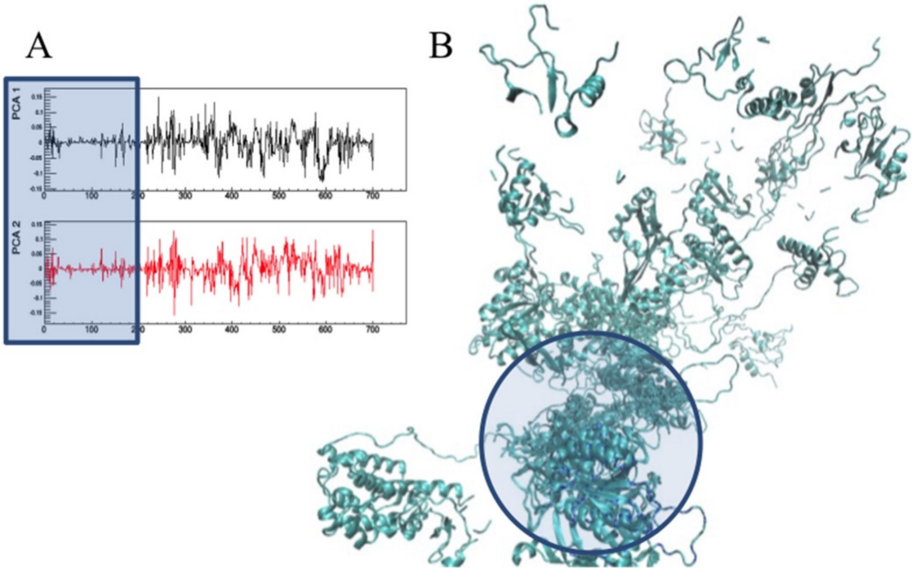

Figure S3. (**A**) Loading plot obtained by processing the PAV profiles of segment B of the ESYN1 sequences of the 13 *Fusarium* isolates, with the selected region of minimal variations highlighted. (**B**) Overlap of the corresponding structural models of segment B, performed according to the positions of the Cα atoms lying in the selected region (highlighted).

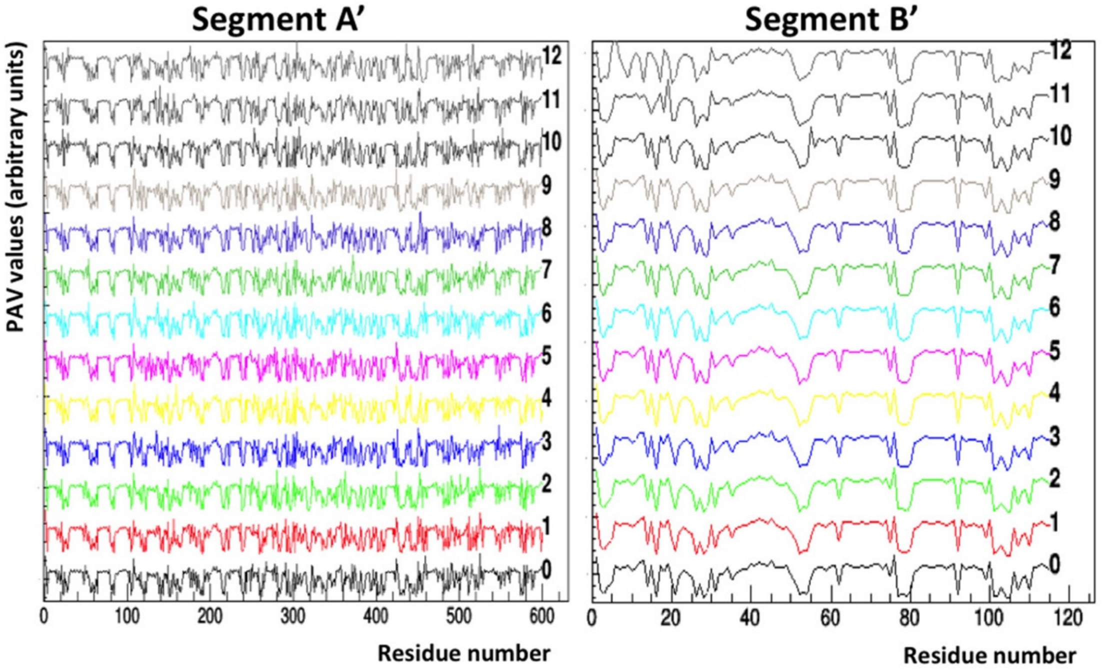

Figure S4. PAV profiles calculated for the ESYN1 sequences of the 13 *Fusarium* isolates, shown separately for segment A′ (left) and B′ (right). The samples' numbering follows that indicated in Table 2.

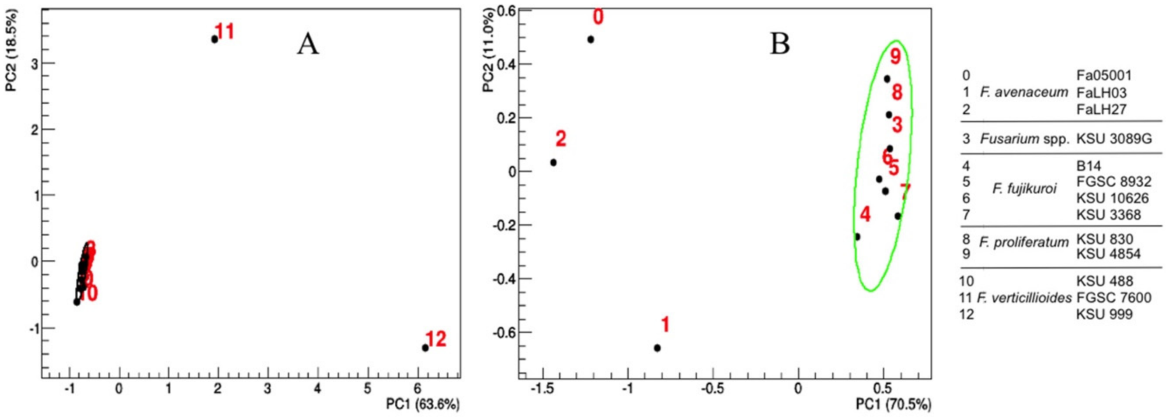

Figure S5. Score plots of the first two principal components (PC1 and PC2) obtained by applicating principal component analysis (PCA) to the segment B′ of (**A**) all 13 ESYN1 sequences of *Fusarium* isolates and of (**B**) the subset constituted by eliminating the *Fusarium verticillioides* strains (Samples 10, 11, 12). The percentage of data variance explained by each principal component is reported on the corresponding axis labels. The 95% confidence level ellipses indicate the results of hierarchical clustering.

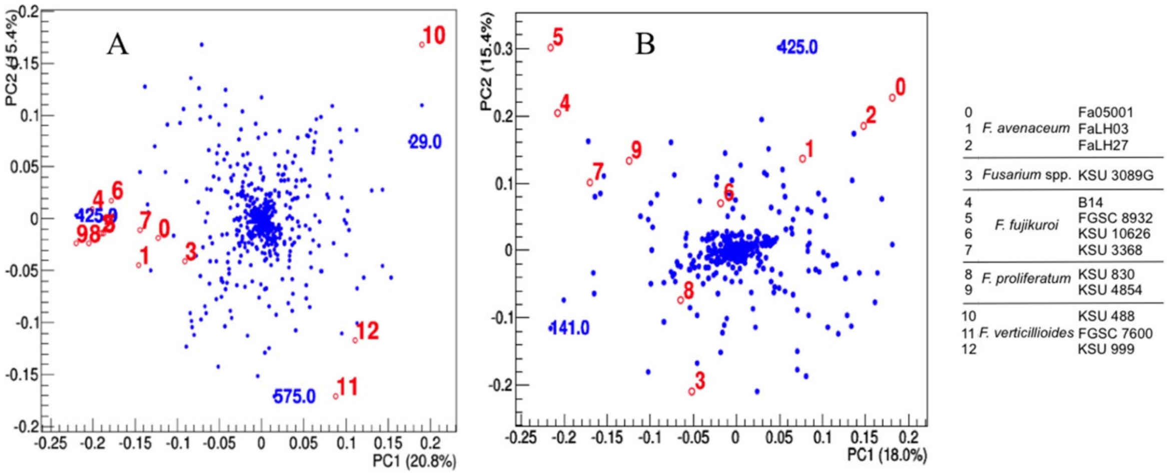

Figure S6. Score (red dots) and loading (blue dots) plots of the first two principal components obtained after application of PCA to the segment A′ of (**A**) all 13 ESYN1 sequences of *Fusarium* isolates and of (**B**) the subset constituted by eliminating the *Fusarium verticillioides* strains (Samples 10, 11, 12). The percentage of data variance explained by each principal component is reported on the corresponding axis labels. The residue number of the loadings more distant from the (0, 0) point is reported.

Table S1. Enniatin and beauvericin production of *Fusarium* strains on chemically-defined production medium (FDM).

Species	Strain	ENN B (μg/kg)	ENN B_1 (μg/g)	ENN A (μg/kg)	ENN A_1 (μg/kg)	BEA (μg/g)
Fusarium fujikuroi	B14	105 ± 16	0.9 ± 0.3	n.d.	n.d.	50 ± 17
Fusarium fujikuroi	FGSC 8932 *	189 ± 60	0.3 ± 0.2	116 ± 84	n.d.	16 ± 20
Fusarium fujikuroi	KSU 10626 *	94 ± 29	0.3 ± 0.1	76 ± 74	n.d.	17 ± 4
Fusarium fujikuroi	KSU 3368 *	151 ± 39	0.6 ± 0.2	43 ± 22	n.d.	50 ± 16
Fusarium verticillioides	FGSC 7600	91 ± 11	0.1 ± 0.1	78 ± 36	n.d.	n.d.
Fusarium verticillioides	ITEM 10027 *	72 ± 12	0.1 ± 0.1	45 ± 20	n.d.	n.d.
Fusarium verticillioides	KSU 488	54 ± 1	n.d.	n.d.	n.d.	n.d.
Fusarium verticillioides	KSU 999	68 ± 3	n.d.	85 ± 84	n.d.	n.d.
Fusarium spp.	KSU 3089G *	44 ± 8	0.1 ± 0.1	n.d.	n.d.	n.d.
Fusarium proliferatum	KSU 830 *	39 ± 3	n.d.	21 ± 23	n.d.	n.d.
Fusarium proliferatum	KSU 4854 *	80 ± 30	0.2 ± 0.1	n.d.	n.d.	4.9 ± 4
Fusarium avenaceum	ITEM 3403	310 ± 57	32 ± 6	n.d.	3 ± 0	0.8 ± 0.4
Fusarium avenaceum	ITEM 3404	230 ± 82	33 ± 12	3 ± 1	3 ± 1	06 ± 0.4

n.d. = not detected; * results confirmed in LC/MS. ENN B = enniatin B; ENN B1 = enniatin B_1; ENN A = enniatin A; ENN A_1 = enniatin A_1; BEA = beauvericin. Detection limit: ENN B = 6 ng/g; ENN B_1 = 7 ng/g; ENN A = 15 ng/g; ENN A_1 = 50 ng/g; BEA = 4 ng/g.

Table S2. Enniatin and beauvericin production of *Fusarium* strains on potato dextrose agar (PDA) medium.

Species	Strain	ENN B (μg/kg)	ENN B_1 (μg/g)	ENN A (μg/kg)	ENN A_1 (μg/kg)	BEA (μg/g)
Fusarium fujikuroi	B14 *	86 ± 58	1.4 ± 0.3	83 ± 57	n.d.	101 ± 15
Fusarium fujikuroi	FGSC 8932 *	478 ± 78	0.7 ± 0.2	575 ± 484	n.d.	100 ± 17
Fusarium fujikuroi	KSU 10626 *	398 ± 220	0.5 ± 0.2	240 ± 217	n.d.	27 ± 8
Fusarium fujikuroi	KSU 3368 *	245 ± 65	0.3 ± 0.1	73 ± 18	n.d.	33 ± 7
Fusarium verticillioides	FGSC 7600 *	244 ± 20	0.2 ± 0.3	168 ± 13	n.d.	n.d.
Fusarium verticillioides	ITEM 10027 *	245 ± 52	0.1 ± 0.1	272 ± 83	n.d.	n.d.
Fusarium verticillioides	KSU 488	144 ± 30	0.1 ± 0.0	222 ± 41	n.d.	n.d.
Fusarium verticillioides	KSU 999	168 ± 12	0.1 ± 0.0	264 ± 63	n.d.	n.d.
Fusarium spp.	KSU 3089G	316 ± 73	0.1 ± 0.0	97 ± 116	n.d.	n.d.
Fusarium proliferatum	KSU 830 *	125 ± 19	0.3 ± 0.0	225 ± 77	n.d.	n.d.
Fusarium proliferatum	KSU 4854	250 ± 26	0.2 ± 0.0	n.d.	n.d.	7.5 ± 3
Fusarium avenaceum	ITEM 3403	383 ± 143	107 ± 36	15 ± 11	9 ± 3	1 ± 2
Fusarium avenaceum	ITEM 3404	122 ± 20	26 ± 9	183 ± 53	3 ± 3	14 ± 6

n.d. = not detected; * results confirmed in LC/MS. ENN B = enniatin B; ENN B_1 = enniatin B_1; ENN A = enniatin A; ENN A_1 = enniatin A_1; BEA = beauvericin. Detection limit: ENN B = 6 ng/g; ENN B_1 = 7 ng/g; ENN A = 15 ng/g; ENN A_1 = 50 ng/g; BEA = 4 ng/g.
